# Triphen­yl(prop-2-yn-1-yl)silane

**DOI:** 10.1107/S1600536812001109

**Published:** 2012-01-18

**Authors:** Björn Nelson, Michaela Schulte, Carsten Strohmann, Hans Preut, Martin Hiersemann

**Affiliations:** a Fakultät Chemie, Technische Universität Dortmund, Otto-Hahn-Strasse 6, 44221 Dortmund, Germany

## Abstract

In the title compound, C_21_H_18_Si, the coordination geometry around the Si atom is a slightly distorted tetra­hedron with C—Si—C angles in the range 106.05 (11) to 110.58 (10) ° and Si–C bond lengths in the range 1.855 (2) to 1.883 (3) Å. The alkyne C—C bond length is 1.167 (4) Å. The dihedral angles between the three phenyl rings are 63.89 (7), 86.38 (7) and 70.51 (8)°. In the crystal, mol­ecules inter­act only by van der Waals forces.

## Related literature

For the first report of the title compound, see: Masson *et al.* (1967 ▶). For background to silane chemistry, see: Abraham *et al.* (2001 ▶, 2003 ▶); Helmboldt & Hiersemann (2003 ▶); Hiersemann (1999 ▶, 2000 ▶); Nelson *et al.* (2011 ▶).
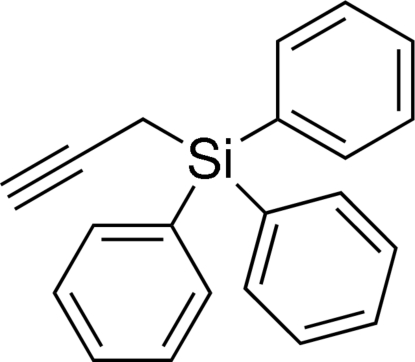


## Experimental

### 

#### Crystal data

C_21_H_18_Si
*M* *_r_* = 298.44
Triclinic, 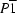

*a* = 9.6668 (11) Å
*b* = 9.6857 (7) Å
*c* = 10.1178 (10) Å
α = 80.289 (7)°
β = 65.189 (10)°
γ = 72.957 (8)°
*V* = 820.98 (16) Å^3^
*Z* = 2
Mo *K*α radiation
μ = 0.14 mm^−1^
*T* = 173 K
0.40 × 0.30 × 0.10 mm

#### Data collection

Oxford Diffraction Xcalibur S CCD diffractometer
Absorption correction: multi-scan (*CrysAlis RED*; Oxford Diffraction, 2008 ▶) *T*_min_ = 0.973, *T*_max_ = 0.986
8081 measured reflections
3224 independent reflections
1940 reflections with *I* > 2σ(*I*)
*R*_int_ = 0.048

#### Refinement

*R*[*F* ^2^ > 2σ(*F* ^2^)] = 0.048
*wR*(*F* ^2^) = 0.086
*S* = 1.05
3224 reflections
211 parameters
H atoms treated by a mixture of independent and constrained refinement
Δρ_max_ = 0.34 e Å^−3^
Δρ_min_ = −0.29 e Å^−3^

### 

Data collection: *CrysAlis CCD* (Oxford Diffraction, 2008 ▶); cell refinement: *CrysAlis CCD*; data reduction: *CrysAlis RED* (Oxford Diffraction, 2008 ▶); program(s) used to solve structure: *SHELXS97* (Sheldrick, 2008 ▶); program(s) used to refine structure: *SHELXL97* (Sheldrick, 2008 ▶); molecular graphics: *SHELXTL-Plus* (Sheldrick, 2008 ▶); software used to prepare material for publication: *SHELXL97* and *PLATON* (Spek, 2009 ▶).

## Supplementary Material

Crystal structure: contains datablock(s) I, global. DOI: 10.1107/S1600536812001109/hb6593sup1.cif

Structure factors: contains datablock(s) I. DOI: 10.1107/S1600536812001109/hb6593Isup2.hkl

Supplementary material file. DOI: 10.1107/S1600536812001109/hb6593Isup3.cml

Additional supplementary materials:  crystallographic information; 3D view; checkCIF report

## Figures and Tables

**Fig. 1.**
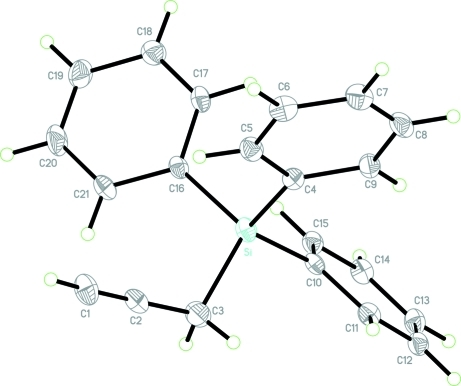
: The molecular structure of the title compound, showing displacement ellipsoids at the 30% probability level.

## supplementary crystallographic information

### Comment

The title compound (I) (Masson *et al.*, 1967) was synthesized from 3-bromoprop-1-yne by Grignard-reaction with ClSiPh_3_. Silane acts as an intermediate *en route* to alkoxy-carbonyl substituted allyl vinyl ethers (Hiersemann, 2000), which exhibit a wide range of reactivity for further synthetic transformation (Hiersemann, 1999; Abraham *et al.*, 2001; Abraham *et al.*, 2003; Helmboldt *et al.*, 2003; Nelson *et al.*, 2011) developed in our laboratory.

### Experimental

To an oven dried, three-necked-flask (equipped with a reflux condenser, a dropping funnel and a stopper, which is ultimately switched with a thermometer) under an atmosphere of argon was added Mg powder (1.02 g, 42 mmol, 2.1 eq) and HgCl_2_ (0.36 g, 1.3 mmol, 0.06 eq). The flask was heated with a heatgun, sealed with a septum and allowed to cool down to room temperature under an atmosphere of argon before Et_2_O (14 ml, 0.33 ml/mmol Mg) was added carefully. The flask was cooled to 273 K and propargylbromide (4.3 ml, 40 mmol, 2 eq, 80% in toluene) in Et_2_O (14 ml, 0.35 ml/mmol bromide) was added dropwise over a period of 25 min. After addition of the first few drops the solution became cloudy and started to boil. The rate of the addition was adjusted to maintain the internal temperature between 273 K and 293 K. The dark solution was stirred further for 50 min at 273 K before chlorotriphenylsilane (5.9 g, 20 mmol, 1 eq) in Et_2_O (50 ml, 2.5 ml/mmol silane) was added dropwise over a period of 30 min. The resulting reaction mixture was allowed to warm to room temperature overnight (16 h) and was then diluted by the careful addition of saturated aqueous NH_4_Cl solution and n-pentane. The aqueous layer was extracted with *n*-pentane (3 × and the combined organic phases were dried (MgSO_4_) and concentrated under reduced pressure (greater than 5 mbar). Purification by flash chromatography (*n*-pentane/Et_2_O 100/1) afforded silane I (2.3 g, 7.7 mmol, 38%) as a white solid. Subsequent recrystallization of I by vapor diffusion technique from isohexane and ethyl acetate provided colourless plates of (I). *R_f_* 0.34 (cyclohexane/ethyl acetate 20/1); ^1^H NMR (CDCl_3_, 400 MHz, δ): 1.87 (t, *J* = 2.9 Hz, 1H), 2.35 (d, *J* = 3.0 Hz, 2H), 7.38–7.48 (m, 9H), 7.59–7.60 (m, 6H); C_21_H_18_Si, *M* = 298.45 g/mol.

### Refinement

The hydrogen atoms of the phenyl rings were placed in calculated positions with C–H bond distances of 0.95Å and refined as riding on their parent atoms with *U*_iso_ = 1.2 *x U*_eq_(C). For the remaining hydrogen atoms coordinates and an isotropic temperature factor were refined.

### Crystal data

**Table:**

C_21_H_18_Si	*Z* = 2
*M**_r_* = 298.44	*F*(000) = 316
Triclinic, *P*1	*D*_x_ = 1.207 Mg m^−^^3^
Hall symbol: -P 1	Mo *K*α radiation, λ = 0.71073 Å
*a* = 9.6668 (11) Å	Cell parameters from 2879 reflections
*b* = 9.6857 (7) Å	θ = 2.2–29.1°
*c* = 10.1178 (10) Å	µ = 0.14 mm^−^^1^
α = 80.289 (7)°	*T* = 173 K
β = 65.189 (10)°	Plate, colourless
γ = 72.957 (8)°	0.40 × 0.30 × 0.10 mm
*V* = 820.98 (16) Å^3^	

### Data collection

**Table:**

Oxford Diffraction Xcalibur S CCD diffractometer	3224 independent reflections
Radiation source: Enhance (Mo) X-ray Source	1940 reflections with *I* > 2σ(*I*)
graphite	*R*_int_ = 0.048
Detector resolution: 16.0560 pixels mm^-1^	θ_max_ = 26.0°, θ_min_ = 2.2°
ω scans	*h* = −11→11
Absorption correction: multi-scan (*CrysAlis RED*; Oxford Diffraction, 2008)	*k* = −11→11
*T*_min_ = 0.973, *T*_max_ = 0.986	*l* = −12→12
8081 measured reflections	

### Refinement

**Table:**

Refinement on *F*^2^	Primary atom site location: structure-invariant direct methods
Least-squares matrix: full	Secondary atom site location: difference Fourier map
*R*[*F*^2^ > 2σ(*F*^2^)] = 0.048	Hydrogen site location: inferred from neighbouring sites
*wR*(*F*^2^) = 0.086	H atoms treated by a mixture of independent and constrained refinement
*S* = 1.05	*w* = 1/[σ^2^(*F*_o_^2^) + (0.0228*P*)^2^] where *P* = (*F*_o_^2^ + 2*F*_c_^2^)/3
3224 reflections	(Δ/σ)_max_ < 0.001
211 parameters	Δρ_max_ = 0.34 e Å^−^^3^
0 restraints	Δρ_min_ = −0.29 e Å^−^^3^

### Special details

**Table:**

Experimental. Absorption correction: CrysAlis RED, Oxford Diffraction Ltd., Version 1.171.32.37 (release 24-10-2008) Empirical absorption correction using sperical harmonics, implemented in SCALE3 ABSPACK scaling algorithm.
Geometry. All e.s.d.'s (except the e.s.d. in the dihedral angle between two l.s. planes) are estimated using the full covariance matrix. The cell e.s.d.'s are taken into account individually in the estimation of e.s.d.'s in distances, angles and torsion angles; correlations between e.s.d.'s in cell parameters are only used when they are defined by crystal symmetry. An approximate (isotropic) treatment of cell e.s.d.'s is used for estimating e.s.d.'s involving l.s. planes.
Refinement. Refinement of *F*^2^ against ALL reflections. The weighted *R*-factor *wR* and goodness of fit *S* are based on *F*^2^, conventional *R*-factors *R* are based on *F*, with *F* set to zero for negative *F*^2^. The threshold expression of *F*^2^ > σ(*F*^2^) is used only for calculating *R*-factors(gt) *etc*. and is not relevant to the choice of reflections for refinement. *R*-factors based on *F*^2^ are statistically about twice as large as those based on *F*, and *R*- factors based on ALL data will be even larger.

### Fractional atomic coordinates and isotropic or equivalent isotropic displacement parameters (Å^2^)

**Table:**

	*x*	*y*	*z*	*U*_iso_*/*U*_eq_	
C1	0.8154 (3)	0.5812 (3)	0.1515 (4)	0.0418 (7)	
C2	0.7846 (3)	0.6191 (2)	0.2662 (3)	0.0332 (6)	
C3	0.7508 (3)	0.6647 (3)	0.4086 (3)	0.0395 (7)	
C4	0.7985 (2)	0.9730 (2)	0.3179 (2)	0.0257 (6)	
C5	0.8267 (2)	0.9789 (2)	0.1710 (2)	0.0328 (6)	
H5	0.8735	0.8920	0.1205	0.039*	
C6	0.7884 (3)	1.1085 (2)	0.0958 (3)	0.0368 (6)	
H6	0.8073	1.1101	−0.0044	0.044*	
C7	0.7222 (3)	1.2353 (2)	0.1697 (3)	0.0382 (7)	
H7	0.6958	1.3246	0.1195	0.046*	
C8	0.6944 (2)	1.2329 (2)	0.3138 (3)	0.0378 (7)	
H8	0.6493	1.3206	0.3631	0.045*	
C9	0.7317 (2)	1.1033 (2)	0.3891 (3)	0.0308 (6)	
H9	0.7118	1.1028	0.4894	0.037*	
C10	0.8073 (2)	0.8192 (2)	0.6057 (2)	0.0256 (5)	
C11	0.6509 (2)	0.8669 (2)	0.7011 (2)	0.0286 (6)	
H11	0.5712	0.8932	0.6635	0.034*	
C12	0.6081 (3)	0.8771 (2)	0.8479 (3)	0.0297 (6)	
H12	0.5003	0.9082	0.9102	0.036*	
C13	0.7231 (3)	0.8417 (2)	0.9041 (3)	0.0314 (6)	
H13	0.6949	0.8494	1.0050	0.038*	
C14	0.8787 (3)	0.7953 (2)	0.8126 (2)	0.0301 (6)	
H14	0.9578	0.7708	0.8509	0.036*	
C15	0.9206 (2)	0.7841 (2)	0.6660 (2)	0.0285 (6)	
H15	1.0285	0.7520	0.6046	0.034*	
C16	1.0757 (2)	0.7216 (2)	0.3174 (2)	0.0247 (5)	
C17	1.1798 (3)	0.8035 (2)	0.2971 (2)	0.0313 (6)	
H17	1.1386	0.9000	0.3275	0.038*	
C18	1.3405 (3)	0.7505 (2)	0.2347 (3)	0.0364 (6)	
H18	1.4086	0.8087	0.2246	0.044*	
C19	1.4016 (3)	0.6110 (2)	0.1867 (3)	0.0417 (7)	
H19	1.5123	0.5735	0.1418	0.050*	
C20	1.3025 (3)	0.5280 (2)	0.2043 (3)	0.0439 (7)	
H20	1.3449	0.4325	0.1712	0.053*	
C21	1.1409 (3)	0.5807 (2)	0.2696 (2)	0.0337 (6)	
H21	1.0738	0.5207	0.2820	0.040*	
H1	0.764 (3)	0.588 (2)	0.475 (3)	0.053 (8)*	
H2	0.646 (3)	0.707 (2)	0.454 (3)	0.049 (8)*	
H3	0.839 (2)	0.557 (2)	0.066 (2)	0.030 (7)*	
Si	0.85982 (7)	0.79610 (6)	0.41079 (7)	0.02875 (19)	

### Atomic displacement parameters (Å^2^)

**Table:**

	*U*^11^	*U*^22^	*U*^33^	*U*^12^	*U*^13^	*U*^23^
C1	0.0559 (19)	0.0359 (16)	0.042 (2)	−0.0082 (13)	−0.0273 (18)	−0.0081 (14)
C2	0.0379 (15)	0.0241 (13)	0.0428 (18)	−0.0094 (11)	−0.0203 (14)	−0.0002 (12)
C3	0.0437 (18)	0.0345 (16)	0.042 (2)	−0.0121 (13)	−0.0166 (16)	−0.0017 (14)
C4	0.0234 (13)	0.0262 (12)	0.0294 (15)	−0.0051 (10)	−0.0131 (12)	−0.0010 (11)
C5	0.0318 (14)	0.0298 (13)	0.0381 (17)	−0.0064 (11)	−0.0155 (13)	−0.0025 (12)
C6	0.0373 (15)	0.0431 (15)	0.0328 (16)	−0.0103 (12)	−0.0193 (13)	0.0060 (13)
C7	0.0341 (15)	0.0307 (14)	0.0495 (19)	−0.0038 (11)	−0.0221 (14)	0.0065 (13)
C8	0.0314 (14)	0.0225 (13)	0.0586 (19)	−0.0033 (10)	−0.0188 (14)	−0.0031 (13)
C9	0.0296 (13)	0.0282 (13)	0.0349 (16)	−0.0050 (10)	−0.0127 (12)	−0.0063 (11)
C10	0.0317 (13)	0.0150 (11)	0.0314 (14)	−0.0049 (10)	−0.0148 (12)	0.0008 (10)
C11	0.0287 (14)	0.0243 (13)	0.0360 (16)	−0.0046 (10)	−0.0175 (13)	−0.0004 (11)
C12	0.0279 (13)	0.0227 (12)	0.0366 (16)	−0.0030 (10)	−0.0118 (12)	−0.0046 (11)
C13	0.0400 (15)	0.0281 (13)	0.0281 (15)	−0.0059 (11)	−0.0156 (13)	−0.0050 (11)
C14	0.0344 (15)	0.0265 (13)	0.0333 (16)	−0.0044 (11)	−0.0195 (13)	−0.0008 (11)
C15	0.0277 (13)	0.0214 (12)	0.0345 (16)	−0.0028 (10)	−0.0126 (12)	−0.0018 (11)
C16	0.0330 (14)	0.0165 (11)	0.0244 (14)	−0.0034 (10)	−0.0139 (12)	0.0017 (10)
C17	0.0359 (15)	0.0203 (12)	0.0355 (16)	−0.0002 (11)	−0.0153 (13)	−0.0048 (11)
C18	0.0364 (15)	0.0315 (14)	0.0398 (17)	−0.0081 (11)	−0.0144 (13)	−0.0003 (12)
C19	0.0339 (15)	0.0321 (15)	0.0471 (19)	0.0036 (12)	−0.0117 (14)	−0.0038 (13)
C20	0.0463 (17)	0.0218 (13)	0.0550 (19)	0.0059 (12)	−0.0190 (15)	−0.0086 (12)
C21	0.0415 (15)	0.0189 (12)	0.0422 (17)	−0.0070 (11)	−0.0187 (14)	0.0002 (11)
Si	0.0332 (4)	0.0224 (3)	0.0325 (4)	−0.0035 (3)	−0.0166 (3)	−0.0020 (3)

### Geometric parameters (Å, °)

**Table:**

C1—C2	1.167 (4)	C11—C12	1.377 (3)
C1—H3	0.85 (2)	C11—H11	0.9500
C2—C3	1.453 (4)	C12—C13	1.386 (3)
C3—Si	1.883 (3)	C12—H12	0.9500
C3—H1	0.93 (2)	C13—C14	1.377 (3)
C3—H2	0.92 (2)	C13—H13	0.9500
C4—C5	1.388 (3)	C14—C15	1.379 (3)
C4—C9	1.402 (3)	C14—H14	0.9500
C4—Si	1.871 (2)	C15—H15	0.9500
C5—C6	1.390 (3)	C16—C17	1.387 (3)
C5—H5	0.9500	C16—C21	1.399 (3)
C6—C7	1.387 (3)	C16—Si	1.860 (2)
C6—H6	0.9500	C17—C18	1.376 (3)
C7—C8	1.366 (3)	C17—H17	0.9500
C7—H7	0.9500	C18—C19	1.387 (3)
C8—C9	1.388 (3)	C18—H18	0.9500
C8—H8	0.9500	C19—C20	1.363 (3)
C9—H9	0.9500	C19—H19	0.9500
C10—C11	1.396 (3)	C20—C21	1.385 (3)
C10—C15	1.399 (3)	C20—H20	0.9500
C10—Si	1.855 (2)	C21—H21	0.9500
			
C2—C1—H3	177.5 (15)	C13—C12—H12	120.2
C1—C2—C3	178.4 (3)	C14—C13—C12	119.5 (2)
C2—C3—Si	116.24 (19)	C14—C13—H13	120.2
C2—C3—H1	113.5 (15)	C12—C13—H13	120.2
Si—C3—H1	107.8 (14)	C13—C14—C15	120.6 (2)
C2—C3—H2	110.3 (15)	C13—C14—H14	119.7
Si—C3—H2	106.5 (14)	C15—C14—H14	119.7
H1—C3—H2	101 (2)	C14—C15—C10	121.3 (2)
C5—C4—C9	117.8 (2)	C14—C15—H15	119.3
C5—C4—Si	119.61 (16)	C10—C15—H15	119.3
C9—C4—Si	122.45 (18)	C17—C16—C21	117.0 (2)
C4—C5—C6	121.8 (2)	C17—C16—Si	120.64 (16)
C4—C5—H5	119.1	C21—C16—Si	122.34 (17)
C6—C5—H5	119.1	C18—C17—C16	122.6 (2)
C7—C6—C5	118.8 (2)	C18—C17—H17	118.7
C7—C6—H6	120.6	C16—C17—H17	118.7
C5—C6—H6	120.6	C17—C18—C19	119.1 (2)
C8—C7—C6	120.6 (2)	C17—C18—H18	120.5
C8—C7—H7	119.7	C19—C18—H18	120.5
C6—C7—H7	119.7	C20—C19—C18	119.8 (2)
C7—C8—C9	120.5 (2)	C20—C19—H19	120.1
C7—C8—H8	119.8	C18—C19—H19	120.1
C9—C8—H8	119.8	C19—C20—C21	121.0 (2)
C8—C9—C4	120.4 (2)	C19—C20—H20	119.5
C8—C9—H9	119.8	C21—C20—H20	119.5
C4—C9—H9	119.8	C20—C21—C16	120.5 (2)
C11—C10—C15	116.7 (2)	C20—C21—H21	119.7
C11—C10—Si	121.16 (16)	C16—C21—H21	119.7
C15—C10—Si	122.11 (17)	C10—Si—C16	110.12 (10)
C12—C11—C10	122.3 (2)	C10—Si—C4	110.58 (10)
C12—C11—H11	118.8	C16—Si—C4	109.44 (9)
C10—C11—H11	118.8	C10—Si—C3	106.05 (11)
C11—C12—C13	119.6 (2)	C16—Si—C3	110.24 (12)
C11—C12—H12	120.2	C4—Si—C3	110.37 (11)
			
C9—C4—C5—C6	−1.0 (3)	Si—C16—C21—C20	−178.13 (17)
Si—C4—C5—C6	−177.71 (16)	C11—C10—Si—C16	−176.80 (16)
C4—C5—C6—C7	0.9 (3)	C15—C10—Si—C16	0.4 (2)
C5—C6—C7—C8	−0.3 (4)	C11—C10—Si—C4	62.13 (19)
C6—C7—C8—C9	−0.2 (4)	C15—C10—Si—C4	−120.69 (17)
C7—C8—C9—C4	0.1 (3)	C11—C10—Si—C3	−57.54 (19)
C5—C4—C9—C8	0.5 (3)	C15—C10—Si—C3	119.65 (19)
Si—C4—C9—C8	177.12 (16)	C17—C16—Si—C10	−67.59 (18)
C15—C10—C11—C12	−1.1 (3)	C21—C16—Si—C10	110.05 (19)
Si—C10—C11—C12	176.23 (17)	C17—C16—Si—C4	54.2 (2)
C10—C11—C12—C13	1.2 (3)	C21—C16—Si—C4	−128.19 (19)
C11—C12—C13—C14	−0.7 (3)	C17—C16—Si—C3	175.74 (17)
C12—C13—C14—C15	0.1 (3)	C21—C16—Si—C3	−6.6 (2)
C13—C14—C15—C10	0.0 (3)	C5—C4—Si—C10	−179.73 (17)
C11—C10—C15—C14	0.5 (3)	C9—C4—Si—C10	3.7 (2)
Si—C10—C15—C14	−176.79 (16)	C5—C4—Si—C16	58.8 (2)
C21—C16—C17—C18	−0.8 (3)	C9—C4—Si—C16	−117.74 (18)
Si—C16—C17—C18	176.96 (17)	C5—C4—Si—C3	−62.7 (2)
C16—C17—C18—C19	1.6 (4)	C9—C4—Si—C3	120.76 (19)
C17—C18—C19—C20	−1.2 (4)	C2—C3—Si—C10	−174.65 (19)
C18—C19—C20—C21	0.0 (4)	C2—C3—Si—C16	−55.5 (2)
C19—C20—C21—C16	0.8 (4)	C2—C3—Si—C4	65.6 (2)
C17—C16—C21—C20	−0.4 (3)		
